# Decoding the Impacts of Mating Behavior on Ovarian Development in Mud Crab (*Scylla paramamosain*, Estampador 1949): Insights from SMRT RNA-seq

**DOI:** 10.3390/biology14101362

**Published:** 2025-10-04

**Authors:** Chenyang Wu, Sadek Md Abu, Xiyi Zhou, Yang Yu, Mhd Ikhwanuddin, Waqas Waqas, Hongyu Ma

**Affiliations:** 1 Guangdong Provincial Key Laboratory of Marine Biotechnology, Shantou University, Shantou 515063, China; 23cywu@stu.edu.cn (C.W.); 19masadek@stu.edu.cn (S.M.A.); 22xyzhou@alumni.stu.edu.cn (X.Z.); 20yyu1@stu.edu.cn (Y.Y.); 2 International Joint Research Center for the Development and Utilization of Important Mariculture Varieties Surrounding the South China Sea Region, Shantou University, Shantou 515063, China; 3 STU-UMT Joint Shellfish Research Laboratory, Shantou University, Shantou 515063, China; 4 Higher Institute Centre of Excellence (HICoE), Institute of Tropical Aquaculture and Fisheries, Universiti Malaysia Terengganu, Kuala Nerus 21030, Malaysia; ikhwanuddin@umt.edu.my

**Keywords:** *Scylla paramamosain*, mating behavior, ovarian development, full-length transcriptome, alternative splicing

## Abstract

Pubertal molting represents a pivotal transition in the life cycle of crustaceans, marking the shift from somatic growth to reproductive development. In mud crabs, mating is known to facilitate this process, yet the molecular mechanisms remain poorly understood. Here, we applied full-length transcriptome sequencing to characterize changes in gene expression and alternative splicing (AS) across post-mating ovarian development. AS analysis revealed extensive transcript diversity, predominantly alternative first exon (AF) and alternative 5′ splice site (A5) events, enriched in genes linked to chromatin remodeling, protein regulation, and metabolism, underscoring AS as a fine-tuning mechanism in ovarian development. Comparative analyses revealed profound molecular reprogramming after mating. In the UM vs. M1 comparison, pathways related to serotonin and catecholamine signaling were enriched, suggesting early neuroendocrine regulation. Serotonin likely promoted, while dopamine inhibited, oocyte maturation, indicating a potential “inhibition–activation” switch. In the UM vs. M3 comparison, pathways associated with oxidative phosphorylation, ATP biosynthesis, and lipid metabolism were upregulated, reflecting heightened energy demands during vitellogenesis. ECM-receptor interaction, HIF-1, and IL-17 signaling pathways further pointed to structural remodeling and tissue regulation. Enhanced antioxidant defenses, including upregulation of SOD2, CAT, GPX4, and GSTO1, highlighted the importance of redox homeostasis. Together, these findings provide the first comprehensive view of transcriptional and splicing dynamics underlying post-mating ovarian maturation in *Scylla paramamosain*, offering novel insights into the molecular basis of crustacean reproduction.

## Simple Summary

In crustaceans, pubertal molting marks the shift from growth to reproduction, and mating promotes ovarian maturation. However, the molecular events driving this transition remain unclear. In this study, we used full-length transcriptome sequencing to investigate gene expression and alternative splicing changes in the mud crab (*Scylla paramamosain*) after mating. Our results showed that mating triggered major transcriptional reprogramming in the ovary. Early responses involved serotonin and dopamine signaling, which act as opposing regulators of oocyte maturation. Later stages were characterized by activation of energy metabolism, lipid mobilization, and extracellular matrix remodeling to support yolk accumulation and oocyte growth. We also observed enhanced antioxidant defense, indicating the importance of maintaining redox balance during rapid ovarian development. These findings provide new molecular insights into the reproductive biology of mud crabs and may support future strategies for improving broodstock management in aquaculture.

## 1. Introduction

The genus *Scylla* includes commercially important aquaculture crab species cultured across Indo-Pacific regions [1,2]. Among the *Scylla* crabs, the mud crab (*Scylla paramamosain*, Estampador 1949), a key species, has emerged as one of the most economically important crustaceans and is widely cultured across China and Vietnam [3,4]. *S. paramamosain* is valued for its rapid growth, high fecundity, and strong market demand. In the year 2023, its production was recorded as 157,012 tons in China, showcasing its significance in crustacean aquaculture [5]. Female crabs are important from both ecological and aquaculture perspectives due to their economic value and reproduction capabilities [6]. In hatcheries, female crabs are the primary source of larval production, while their developed ovaries make them highly sought in seafood markets [7,8]. Additionally, their reproductive traits serve as sensitive indicators of environmental stress, making female crabs key models in environmental and molecular research [9]. Ovarian development is a complex process regulated by the neuroendocrine network involving various hormones and enzymes such as ecdysone and vitellogenin [10]. Ovarian development in female crabs after mating demands a higher energy and material supply than unmated individuals [11]. This process involves a multifaceted interplay of molecular entities, including genes, hormones, enzymes, lipids, and proteins, forming a complicated interactive network [12]. However, the molecular profiling of post-mated female mud crabs is largely unexplored, leaving a critical gap in the literature on how mating behavior influences the molecular dynamics of ovarian development.

In mud crabs, ovarian development follows a gradual process consisting of five stages: starting from the undeveloped phase (Stage I), progressing through pre-vitellogenic (Stage II), early and late vitellogenic stages (Stages III and IV), and culminating in the fully mature stage (Stage V) [13]. Before undergoing pubertal molt and mating, mud crabs usually have ovaries at Stage II, which are still small and appear translucent to milky white [14]. In the early stages of ovarian development, oogonia actively proliferate and then begin to differentiate into primary oocytes [15]. As Stage II progresses, follicular cells begin to proliferate around the cytoplasm of the developing oocyte [16]. Following mating, the mud crab undergoes rapid ovarian development and yolk accumulation [17]. Thus, mating behavior serves as a potential catalyst of ovary development and vitellogenesis in female crabs following pubertal molt [18]. Eubrachyuran crabs exhibit six mating strategies, differentiated by the timing of mating relative to molting (soft- or hard-shell), growth pattern (determinate or indeterminate), and seminal receptacle position (dorsal or ventral) [19]. The mud crabs mate during their soft-shell stage and has dorsal seminal receptacles. In the genus *Scylla*, mating involves courtship, extended guarding, sperm plugs, and the absence of hinged vulval coverings [20].

Next-generation sequencing (NGS) has made it possible to quickly and accurately analyze genetic material, opening new doors for understanding how genes function and interact [21,22]. NGS has enabled fast, affordable DNA sequencing, driving the development of tools like RNA-seq, exome sequencing, ChIP-seq, miRNA-seq, RAD-seq, and small RNA sequencing to tackle diverse biological [23,24,25]. However, short-read NGS poses limitations for accurate transcript reconstruction in eukaryotes due to complex splicing and transcript isoforms [26]. This complexity stems from the ability of a single gene to produce multiple isoforms through alternative transcription start sites and RNA processing. Long-read sequencing overcomes this limitation by capturing full-length isoforms, enabling more accurate transcriptome analysis [27]. SMRT RNA-seq produces highly accurate long reads, enabling analysis of alternative splicing, polyadenylation, genome annotation, fusion transcripts, isoform phasing, and long noncoding RNAs [28]. Its real-time, PCR-free workflow minimizes bias, reduces costs, and lowers computational requirements [29]. Full-length transcripts refine genome annotation by precisely mapping gene structures, regulatory elements, and coding regions, facilitating detailed isoform analysis across the transcriptome [30].

In *Portunus trituberculatus*, the GSI of mated females was significantly higher than that of unmated females. Histological observations revealed the presence of exogenous vitellogenic oocytes in mated females, whereas the predominant oocytes in unmated females were previtellogenic oocytes and endogenous vitellogenic oocytes [31]. Previously, Yu et al. [32] reported that ovarian development in unmated mud crabs is prolonged, taking up to 60 days with smaller oocytes, while mated females reach stage III (proliferative stage) within 23 days and develop larger oocytes, indicating that mating behavior triggers ovarian maturation. However, the molecular mechanism linking mating behavior to ovarian development remains unclear. In this study, we employed full-length transcriptome analysis using SMRT RNA-seq to explore differentially expressed genes, alternative splicing events, and regulatory pathways involved in ovarian development in *Scylla paramamosain* before and post-mating stages. These findings provide novel insights into the molecular mechanisms by which mating behavior regulates ovarian maturation in *Scylla paramamosain*. These insights are expected to advance fundamental research on reproductive regulation and to inform the development of improved broodstock management strategies in aquaculture.

## 2. Materials and Methods

### 2.1. Crabs Collection and Mating

A total of 80 female and 30 male mud crabs were collected from a local seafood market in Chaozhou city (23.26° N, 116.54° E) of Guangdong Province in China, with detail information in our previous research [31]. Briefly, crabs were reared individually in water tanks containing filtered seawater maintained at 29 °C and fed twice daily with clams (*Sinonovacula constricta*). Pubertal molting and courtship were monitored using a real-time video surveillance system [33]. Artificial mating between male and female crabs was carried out, following the method of Fazhan et al. [17]. Immediately after reproductive molt, female crabs were paired with potential males in dark and oxygenated mating boxes for 1–2 days to facilitate copulation. Clean water and food were provided to maintain water quality and reduce the risk of male cannibalism during mating. To ensure consistency in the artificial mating process, all pairings were conducted under identical environmental conditions. The procedures were standardized and carried out by the same operator to minimize variation among groups. Each female crab was used only once for mating, while the male crabs were used repeatedly. Additionally, four experimental groups were established to represent key stages of reproductive development: (1) BM—before pubertal molting, (2) UM—unmated females exhibiting courtship behavior without successful copulation, (3) M1—females at 1-day post-mating, and (4) M3—females at 3 days post-mating. Mating was confirmed by detecting spermatophores in the seminal receptacles of female crabs anesthetized on ice [34,35]. The crabs were then dissected, and approximately 50 mg of ovarian tissue was collected and preserved in RNA Keeper Tissue Stabilizer (Vazyme Biotech Co., Ltd., Nanjing, China). Following the incubation at 4 °C for 24 h, the tissue samples were stored −80 °C until RNA sequencing. A total of 12 ovarian tissue samples—three biological replicates each from the BM, UM, M1, and M3 groups—were subjected to RNA sequencing.

### 2.2. RNA Extraction and Sequencing

Total RNA was extracted using the TRIzol Reagent kit (ComWin Biotech, Guangzhou, China). RNA quality was assessed by measuring concentration and integrity using an Agilent 2100 Bioanalyzer (Agilent Technologies, CA, USA), and a Nanodrop 2000 (Agilent Technologies, CA, USA). Only high-quality RNA samples with a RNA integrity number (RIN) ≥ 8 and a 28S/18S ratio ≥ 1.5 were selected for subsequent library construction. For SMRT sequencing, libraries were constructed and sequenced using the PacBio Sequel platform (v Sequel II). Briefly, PacBio single-molecule long reads were processed using RS_IsoSeq (v2.3) in the SMRT Analysis package (v2.3.0.140936.p4.150482) via command line to obtain insert reads. Full-length transcript characterization was performed using the pbtranscript.py script from the same package. The SMRT library was constructed from pooled RNA extracted from all 12 crab ovarian tissue samples. PacBio single-molecule long reads were processed using RS_IsoSeq (v2.3) in the SMRT Analysis package (v2.3.0.140936.p4.150482) via command line to obtain insert reads. Full-length transcript characterization was performed using the pbtranscript.py script from the same package. The Clontech kit was used to identify the 5′ and 3′ primers, with the poly(A) tail upstream of the 3′ primer serving as a key signal for identifying strand-specific full-length reads. Sequencing errors in consensus reads were corrected using LSC 2.0 (https://github.com/Augroup/LSC, accessed on 17 August 2025) with Illumina short reads derived from ovarian tissues of *S. paramamosain* (parameters: runLSC.py -long_reads SQ_SMRT.fa -short_reads SQ_Illumina.fa -output output). The completeness of the SMRT-based transcriptome assembly was then evaluated using DETONATE and Ex90N50 metrics. For Illumina sequencing, three libraries were prepared per group (BM, UM, M1, and M3) and sequenced on the Illumina HiSeq 2500 platform (v 6000) to generate 150 bp paired-end reads. Library construction and sequencing were carried out by Beijing Biomarker Technologies Co., Ltd., Beijing, China.

### 2.3. SMRT Sequencing Data Processing

To identify transcript isoforms, we processed the subread sequence data using a structured bioinformatics pipeline. Firstly, circular consensus sequences (CCS) were generated from the raw subreads using ccs (v4.2.0) with parameters set to -minLength 50, -maxLength 15,000, and -minPasses 1 to ensure high-quality reads. Next, full-length (FL) reads were obtained by removing primers and demultiplexing with lima (v1.11.0), applying the -dump-clips and -peek-guess parameters for accurate read processing. To eliminate noise, FL reads were refined using the isoseq3 (v3.3.0) refine module, requiring polyadenylation (-require-polya) and a minimum poly(A) tail length of 20 nucleotides (-min-polya-length 20). These filtered reads were then clustered into unpolished transcript consensus sequences using the same IsoSeq3 refine module with default settings. Finally, the unpolished transcripts were polished using the isoseq3 polish module (default parameters) to generate high- and low-confidence transcript isoforms, ensuring robust isoform identification for downstream analysis.

### 2.4. Collapsing Redundant Transcripts Isoforms

To reduce redundancy among transcript isoforms, we implemented a two-step collapsing approach. Firstly, high-quality isoforms were aligned to the *Scylla paramamosain* reference genome [36] using minimap2 (v2.18) (default parameters), generating SAM files [37]. Redundant mapped isoforms were then collapsed using cDNA_Cupcake (https://github.com/Magdoll/cDNA_Cupcake) with parameters (-min_aln_coverage 0.95, -min_aln_identity 0.85, -dun-merge-5-shorter). For unmapped transcripts, we employed Cogent (v8.0) (https://github.com/Magdoll/Cogent) and cDNA_Cupcake (default parameters) to identify distinct gene families. A “fake genome” was constructed by concatenating Cogent-derived contigs, allowing unmapped transcripts to be realigned and collapsed following the same criteria. Finally, CD-HIT (v4.8.1) [38] was applied to both mapped and unmapped isoforms to remove highly similar sequences (identity threshold: 95%), ensuring a non-redundant transcriptome for downstream analysis.

### 2.5. Completeness and Characteristics Analysis of Reconstructed Transcriptomes

To evaluate the quality and completeness of the full-length transcriptomes, we conducted benchmarking universal single-copy orthologs (BUSCO) (v5.1.3) [39] analysis in transcriptome mode using the Arthropoda lineage dataset (arthropoda_odb10) to assess the representation of evolutionarily conserved single-copy orthologs [40]. The full-length transcripts were then systematically classified by comparing them to the reference genome annotation using gffcompare (v0.12.2) (http://ccb.jhu.edu/software/stringtie/gffcompare.shtml) [41] (Table 1). To identify shared and unique transcripts across the datasets, we performed pairwise comparisons using BLASTn (v2.11.0^+^) with stringent parameters (*−e* value ≤ 1 × 10^−10^ and percent identity ≥ 95%), where each transcriptome was alternately used as both the reference database and query sequence. This comprehensive analytical approach enabled us to thoroughly characterize the transcriptomes and identify both conserved and sample-specific transcripts.

### 2.6. Gene Functional Annotation

To elucidate the biological functions of the full-length transcripts, we performed comprehensive gene functional annotation through a multi-step bioinformatics. Firstly, we employed TransDecoder to identify potential open reading frames (ORFs) from the transcripts, selecting the first ORF when multiple candidates were present in a single transcript. These ORFs were then functionally annotated using eggNOG-mapper (v2.1.4) [42] to obtain classifications from multiple databases, including Clusters of Orthologous Groups (COG), Gene Ontology (GO), Kyoto Encyclopedia of Genes and Genomes (KEGG), and Protein families database (Pfam). To further validate and expand the functional annotations, we conducted homology searches against four major protein databases (Swiss-Prot, TrEMBL, UniRef90, and NR) using DIAMOND (v2.0.4.142) with stringent parameters (blastp, -outfmt 6, -max_target_seqs 1, -*e* value 1 × 10^−5^). Finally, all annotation results were consolidated into a comprehensive, tab-delimited summary file to facilitate further functional analyses.

### 2.7. Alternative Splicing (AS) Events Analysis

Alternative splicing (AS) events were analyzed using SUPPA2 software (v 2.4) [43], and seven major types of AS events were identified using the generateEvents function of SUPPA2 with default parameters. These included skipped exon (SE), mutually exclusive exon (MX), alternative 5′ splice site (A5), alternative 3′ splice site (A3), retained intron (RI), alternative first exon (AF), and alternative last exon (AL). The distribution and frequency of these AS events were compared across different mating groups to identify potential splicing differences linked to genetic background. Furthermore, Gene Ontology (GO) enrichment analysis of genes associated with AS events was conducted using the clusterProfiler package (v 4.0.4), based on functional annotations obtained through eggNOG-mapper.

### 2.8. Quantification of Identified Transcripts

To investigate differential alternative splicing (AS) events among the BM, UM, M1, and M3 groups, subread sequences from each group were processed to reconstruct group-specific transcriptomes, following the pipeline previously described. Transcript and gene expression quantification were then performed using Salmon (mapping-based mode) [44]. Firstly, a reference transcriptome index was generated by constructing a k-mer hash (k = 31) from the reconstructed transcript sequences. Subsequently, clean RNA-seq reads were aligned to the corresponding group-specific transcriptomes using Salmon’s quant module with the parameters -l IU and -validateMappings for fast and accurate alignment. Finally, transcript abundance was estimated using the quantmerge module, which produced transcript per million (TPM) values for each sample. These TPM values were subsequently used to calculate percent spliced-in (PSI) values, providing a quantitative measure of alternative splicing across the different groups.

### 2.9. Differential Alternative Splicing (DAS) Events, Differential Expressed Transcripts (DETs) and Their Enrichment Analysis

To identify differential alternative splicing (DAS) events between four groups (BM, UM, M1, and M3), PSI values for each AS event were calculated using the psiPerEvent function of SUPPA2, based on the transcript per million (TPM) values and the corresponding AS event annotations. Differential splicing analysis was then performed using the diffSplice function in SUPPA2, with statistical significance defined by a threshold of |ΔPSI| ≥ 0.15 and *p* < 0.05. In parallel, differential expression analysis of transcripts between groups (BM, UM, M1, and M3) was conducted using DESeq2 (v 1.32.0) [45], based on TPM. Differentially expressed transcripts (DETs) were identified using the cutoff |log_2_(fold change)| ≥ 1 and *p* < 0.05. To explore the functional implications of both significant DAS events and DETs, Gene Ontology (GO) and KEGG pathway enrichment analyses were performed. Gene annotations were first retrieved from eggNOG-mapper results, and enrichment analysis was carried out using clusterProfiler [46]. Only GO terms or KEGG pathways with *p* < 0.05 were considered significantly enriched.

### 2.10. Validation of Differentially Expressed Genes

To validate the transcriptome sequencing results, five differentially expressed genes were selected for quantitative real-time PCR (qRT-PCR). Specific primers for the selected five genes were designed using Primer Premier 5.0. To validate differential gene expression between (BM, UM, M1, and M3), two qPCR kits, including the miRcute Plus miRNA qPCR Kit (SYBR Green) and the Talent qPCR Premix (SYBR Green) (both from TIANGEN Biotech, Beijing, China), were used to perform qRT-PCR for the five selected genes based on a LightCycler^®^ 480 system (Roche Applied Science, Indianapolis, IN, USA). 18S rRNA served as the internal control (reference gene). Each gene was amplified in three biological replicates and three technical replicates. Relative expression levels were calculated using the 2^−ΔΔCt^ method [47]. Statistical significance was determined using a Student’s *t*-test (*p* < 0.05) implemented in R software (v 4.1.0).

## 3. Results

### 3.1. Summary of PacBio Iso-Seq Data and Collapsing Redundant Isoforms

In the present study, PacBio SMRT RNA-seq was performed on RNA samples obtained from four experimental groups (BM, UM, M1, and M3), generating a total of 59,566,665 subreads (approximately 34.0 GB) and 880,044 circular consensus sequences (CCSs).

Following IsoSeq3 refinement, clustering, and polishing, a total of 59,156 high-quality isoforms (average length: 2074.8 bp) and 36 low-quality isoforms (average length: 2293.1 bp) were obtained. Given their minimal proportion, the low-quality isoforms were excluded from subsequent analyses (Table 2). Reference-guided collapsing of redundant transcripts yielded 50,170 unique isoforms. Additionally, pseudo-reference genome construction identified 1483 novel isoforms. The final integration of isoforms using CD-HIT resulted in a non-redundant transcriptome comprising 51,637 isoforms, with an average length of 2056 bp (Table 3).

### 3.2. Evaluation of Reconstructed Transcriptomes

**Figure 1:**
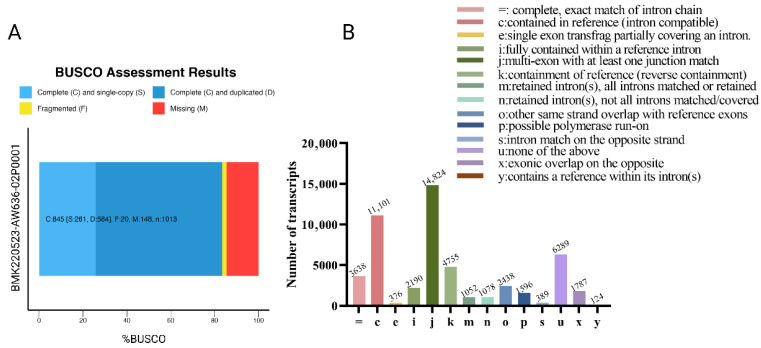
The completeness and characteristics analysis of reconstructed transcriptomes. (**A**) BUSCO assessment results of collapsed redundant transcripts. The Y-axis represents the reconstructed transcriptome. Both the X-axis and the different colors of the box represent the proportion of different categories, including complete and single-copy, complete and duplicated, fragmented, or missing. (**B**) The comparison of reconstructed transcriptomes with reference genome annotation using gffcompare v0.12.2 software. The Y-axis represents the number of genes. The X-axis and the different colored bars represent different categories.

In this study, the completeness and structural characteristics of the reconstructed transcriptome were comprehensively evaluated (Figure 1). Using BUSCO analysis, we identified 25.8% complete and single-copy orthologs (*n* = 261), 57.7% complete and duplicated (*n* = 584), 1.9% fragmented (*n* = 20), and 14.6% missing orthologs (*n* = 148) (Figure 1A), indicating a reasonably complete representation of conserved gene content. Comparative analysis with the reference genome annotation revealed that the reconstructed transcriptome comprised 11,101 transcripts matching known isoforms (category “c”), 14,824 potentially novel isoforms sharing splice junctions with known genes (category “j”), and 6289 entirely novel transcripts from previously unannotated loci (category “u”) (Figure 1B). These findings suggest that the reconstructed transcriptome not only recovers a substantial proportion of the reference annotation but also uncovers a considerable number of novel and potentially functionally relevant isoforms.

### 3.3. Functional Annotation

**Figure 2:**
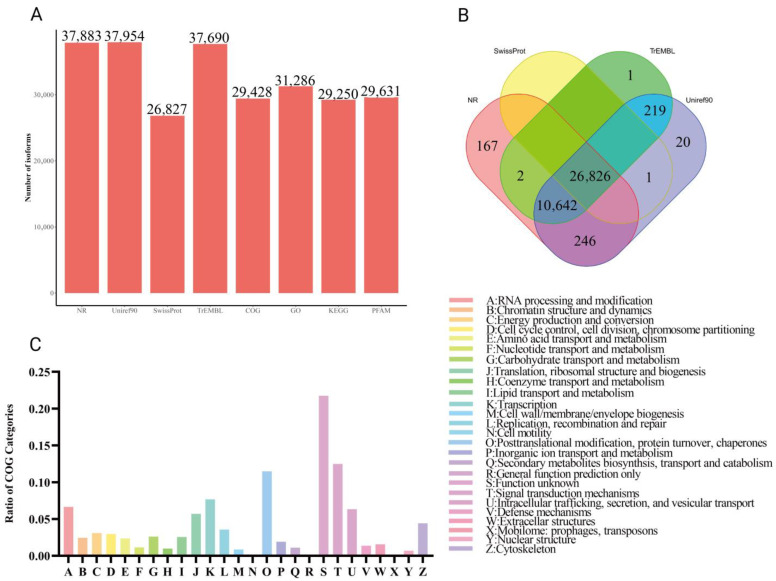
The summary of gene functional annotation using different databases. (**A**) Statistics of isoforms annotation results using different databases, including NR, Uniref90, Swiss-Prot, TrEMBL, COG, GO, KEGG, and PFAMs. The Y-axis represents the number of annotated isoforms. The X-axis represents different databases. (**B**) Venn diagrams showing the overlapping isoforms annotation results obtained using a different database. (**C**) COG profiles of transcript isoforms.

To gain biological insight into the reconstructed transcriptome, functional annotation was performed and aligned with multiple well-established databases. The number of transcript isoforms annotated varied across different databases, ranging from 26,827 (Swiss-Prot) to 37,883 (TrEMBL), demonstrating broad but variable annotation coverage (Figure 2A). The overlap in annotation results across four databases—Swiss-Prot, TrEMBL, UniRef90, and NR. A total of 38,124 transcript isoforms were annotated in at least one of these databases, among which 26,826 isoforms were commonly identified by all four, reflecting a high degree of annotation consistency and supporting the reliability of the reconstructed transcriptome (Figure 2B). To further characterize transcript functions, isoforms were classified into Clusters of Orthologous Groups (COG) categories, as presented in Figure 2C. The largest group of transcripts was assigned to Category S (Function unknown), followed by Category T (Signal transduction mechanisms) and Category O (Posttranslational modification, protein turnover, chaperones). This functional distribution suggests the transcriptome encompasses a wide range of cellular roles, with a substantial portion representing uncharacterized or potentially novel functions.

### 3.4. Alternative Splicing Events

**Figure 3:**
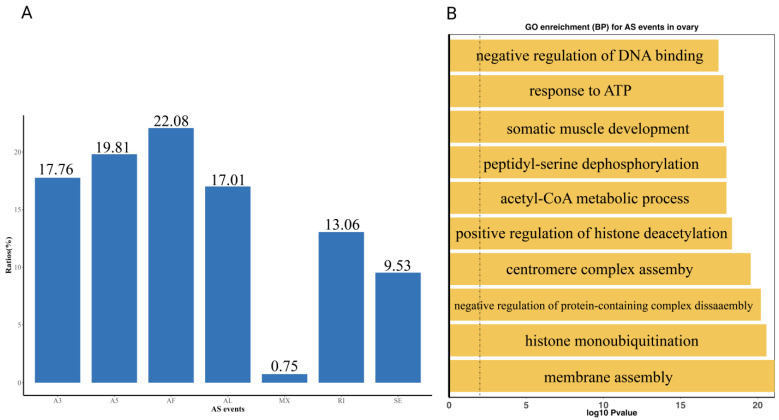
The summary overview of variable splicing events in the reconstructed transcript. (**A**) The proportion of each AS type. The Y-axis represents the proportion of different AS events. The X-axis represents different AS event types, including SE (skipped exon), MX (mutually exclusive exon), A5 (alternative 5′ splice site), A3 (alternative 3′ splice site), RI (retained intron), AF (alternative first exon), and AL (alternative last exon). (**B**) The top ten significantly biological processes (BP) obtained from Gene Ontology (GO) enrichment analysis using genes with AS events in the reconstructed transcript. The Y-axis represents different BP categories. The X-axis represents the corresponding −log_10_ transformed *p*-value.

To further characterize transcriptomic complexity, we investigated alternative splicing (AS) events in the reconstructed transcriptome. A total of seven distinct AS event types were identified: alternative 3′ splice site (A3), alternative 5′ splice site (A5), alternative first exon (AF), alternative last exon (AL), mutually exclusive exon (MX), retained intron (RI), and skipped exon (SE). A detailed analysis of AS patterns in ovarian tissue revealed that AF (alternative first exon) was the most prevalent event, followed by A5, A3, AL, RI, SE, and MX (Figure 3A; Table 4 and Table S1). To elucidate the biological significance of these splicing events, Gene Ontology (GO) enrichment analysis was performed on all genes undergoing alternative splicing. As shown in Figure 3B, the top ten significantly enriched biological process (BP) terms were predominantly associated with protein regulation and chromatin remodeling. These included peptidyl-serine dephosphorylation, positive regulation of histone deacetylation, and histone monoubiquitination, suggesting that alternative splicing in ovarian tissue may contribute substantially to post-translational modification and epigenetic control mechanisms.

### 3.5. Differential Alternative Splicing (DAS) Events, Differential Expressed Transcripts (DETs), and Their Differential Expression Analysis

**Figure 4:**
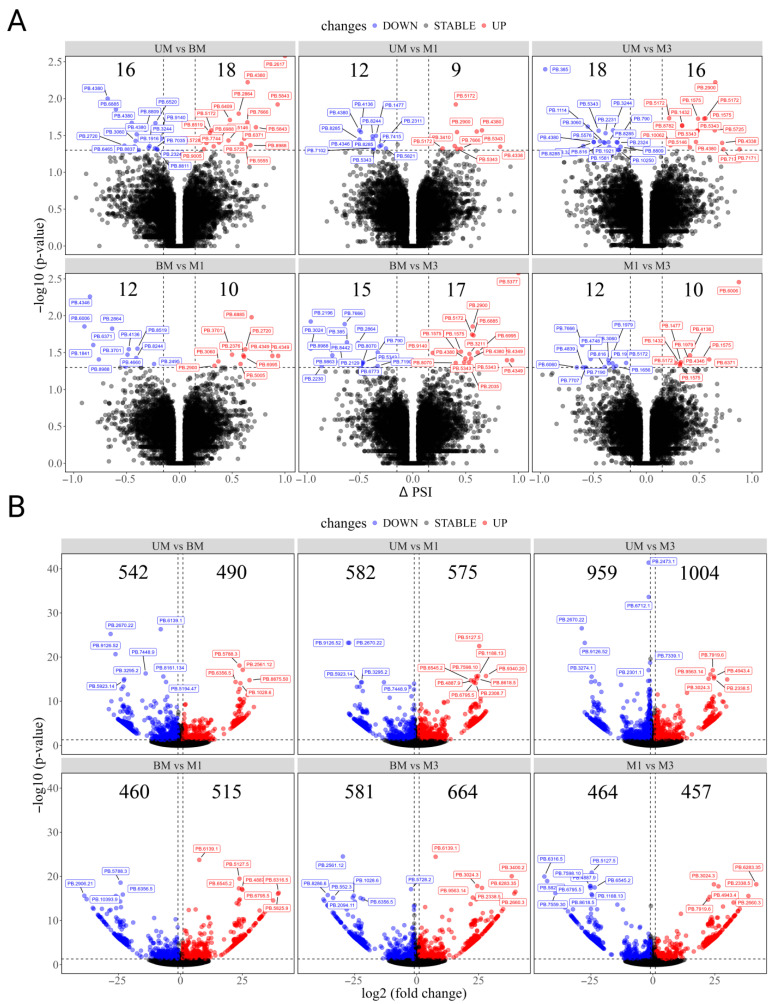
Differential expression analysis among different groups. BM—before pubertal molting, UM—unmated females exhibiting courtship behavior without successful copulation, M1—females at 1-day post-mating, and M3—females at 3 days post-mating. (**A**) The volcano plot indicates *p*-values with minus log_10_-transformed for AS events (Y-axis) against their corresponding difference in inclusion levels (ΔPSI) of each AS event (X-axis). The horizontal gray dotted line represents the significant threshold (0.05). The red, blue, and gray points represent up-regulated, down-regulated, and non-regulated AS events in groups, respectively. (**B**) The volcano plot indicates with minus log_10_-transformed for genes (Y-axis) against their corresponding log_2_(|fold change|) of echo gene (X-axis).

To better understand transcriptomic differences between experimental groups, we examined both differential alternative splicing (DAS) events and differentially expressed transcripts (DETs) across the BM, UM, M1, and M3 samples. The DAS analysis revealed that the number of significant splicing events varied among the six pairwise comparisons. Most of these events were linked to protein-coding genes and were dominated by four splicing types: retained intron (RI), alternative 5′ splice site (A5), alternative 3′ splice site (A3), and alternative first exon (AF) (Table 5; Figure 4A; Table S2). In terms of differential expression, the UM vs. M3 comparison showed the largest number of significantly different transcript isoforms (1963), while M1 vs. M3 had the fewest (921) (Table 5; Figure 4B; Table S3). Interestingly, the most highly differentially expressed transcripts (based on *p*-value) varied depending on the comparison group. For instance, in the UM vs. M1 group, notable transcripts included PB.3431.2, PB.10089.7 (with domains found in myosin and kinesin tails), PB.10152.5, PB.552.5 (a member of the Rho family of small GTPases), and PB.9323.2. In the UM vs. M3 group, top transcripts included PB.8738.1 (belonging to the uncharacterized UPF0029 protein family), along with PB.5936.9, PB.229.2, PB.9078.1, and PB.9829.3. For the M1 vs. M3 comparison, prominent transcripts included PB.5147.8 (containing a BRIX domain), PB.6586.23 (with a VWC_def domain), PB.2909.2 (from the glycosyl hydrolase family 31), PB.6759.12 (from the class-I aminoacyl-tRNA synthetase family), and PB.705.6 (a DnaJ homolog subfamily B member).

### 3.6. DAS and DETs Enrichment Analysis

**Figure 5:**
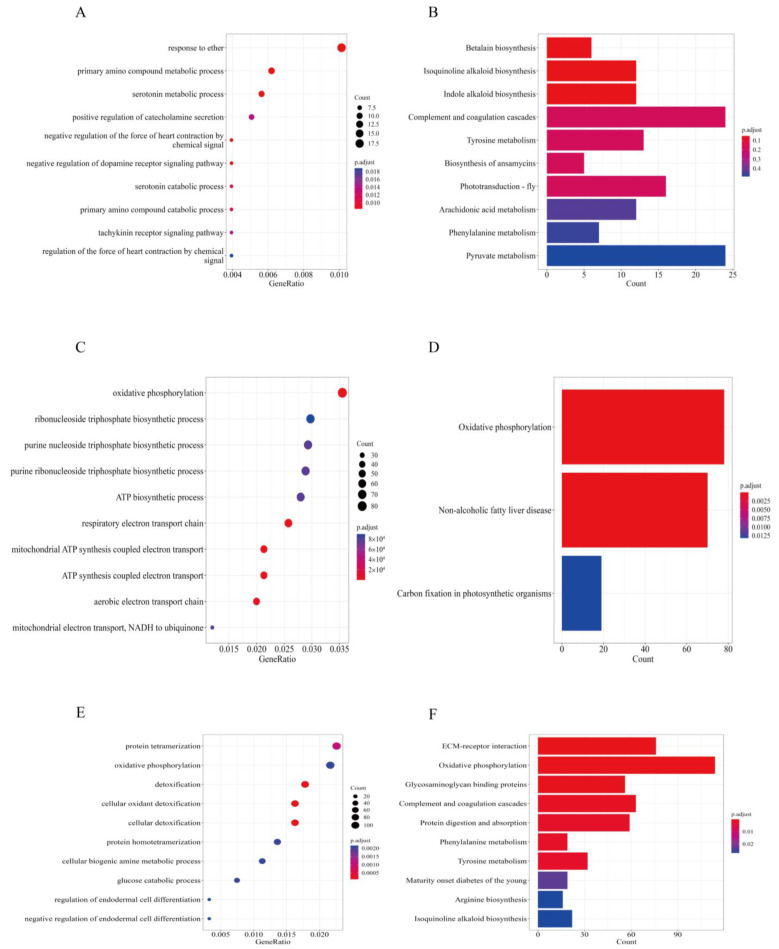
Results of GO and KEGG analyses. (**A**) The top ten significant gene ontology (GO) terms obtained from GO enrichment analysis using genes with DAS events or DETs in UM (unmated females exhibiting courtship behavior without successful copulation) vs. M1 (females at 1-day post-mating). The Y-axis represents different GO term categories. The X-axis represents the proportion of significantly expressed genes in the list of corresponding GO terms (GeneRatio). Different sizes and colors of circles represent the number of significantly expressed genes and the corresponding adjusted *p*-value of GO terms. (**B**) The top ten significant pathways obtained from KEGG enrichment analysis using genes with DAS events or DEGs in UM vs. M1. The Y axis represents different pathway categories. The X-axis represents the number of significantly expressed genes in the corresponding pathway. Different colors represent the different adjusted *p*-values of the pathway. (**C**) The top ten significant gene ontology (GO) terms obtained from GO enrichment analysis using genes with DAS events or DETs in UM (unmated females exhibiting courtship behavior without successful copulation) vs. M3 (females at 3 days post-mating). (**D**) The top ten significant pathways obtained from KEGG enrichment analysis using genes with DAS events or DEGs in UM vs. M3. (**E**) The top ten significant gene ontology (GO) terms obtained from GO enrichment analysis using genes with DAS events or DETs in six groups. (**F**) The top ten significant pathways obtained from KEGG enrichment analysis using genes with DAS events or DEGs in six groups.

To gain deeper insights into the molecular mechanisms underlying transcriptomic changes, both Gene Ontology (GO) and Kyoto Encyclopedia of Genes and Genomes (KEGG) enrichment analyses were performed using genes associated with differentially expressed transcripts (DETs) and differentially alternative splicing (DAS) events. In the UM vs. M1 comparison, the top ten significantly enriched GO biological process terms were primarily linked to signaling pathway activation and neurochemical processes. These included primary amino compound metabolic processes, serotonin metabolism, positive regulation of catecholamine secretion, and negative regulation of cardiac contractility via chemical signaling (Figure 5A).

KEGG pathway analysis further revealed enrichment in biosynthesis and metabolic pathways, such as betalain biosynthesis, isoquinoline alkaloid biosynthesis, indole alkaloid biosynthesis, arachidonic acid metabolism, phenylalanine metabolism, and pyruvate metabolism (Figure 5B). In the UM vs. M3 comparison, GO enrichment pointed predominantly to pathways involved in energy metabolism, including oxidative phosphorylation, ribonucleoside triphosphate biosynthetic process, ATP biosynthetic process, respiratory electron transport chain, and mitochondrial ATP synthesis coupled with electron transport (Figure 5C). The KEGG results highlighted oxidative phosphorylation, non-alcoholic fatty liver disease (NAFLD), and carbon fixation in photosynthetic organisms as the most enriched pathways (Figure 5D). A broader integrative analysis of DAS events and DETs across all six group comparisons revealed a recurring enrichment in functional categories related to cellular energy production, protein complex formation, detoxification, and developmental regulation. The top GO terms included protein tetramerization, oxidative phosphorylation, cellular detoxification, biogenic amine metabolism, glucose catabolic process, and regulation of endodermal cell differentiation (Figure 5E). Correspondingly, KEGG pathway enrichment across comparisons indicated consistent involvement in pathways such as ECM–receptor interaction, oxidative phosphorylation, glycosaminoglycan binding, complement and coagulation cascades, protein digestion and absorption, phenylalanine metabolism, tyrosine metabolism, maturity-onset diabetes of the young, arginine biosynthesis, and isoquinoline alkaloid biosynthesis (Figure 5F). These findings collectively underscore the interplay between transcriptomic regulation, metabolic activity, and tissue-specific functional remodeling.

### 3.7. Validation of Significant Differential Expression Transcripts

**Figure 6:**
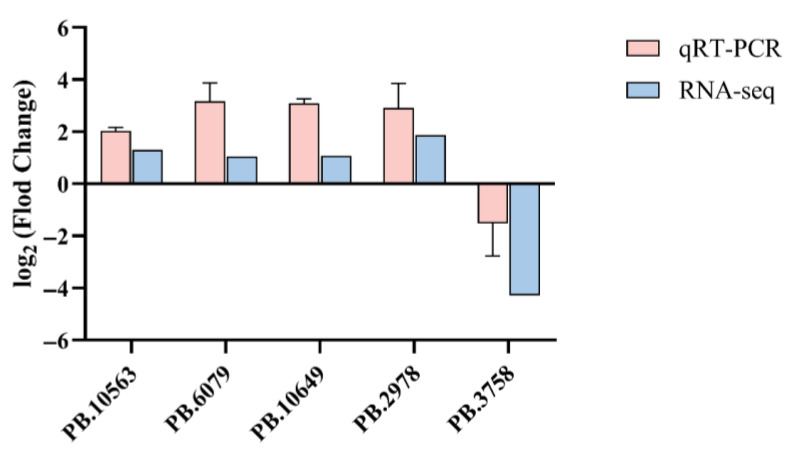
Validation of significantly differentially expressed genes. The Y-axis is log_2_ Flod Change, X-axis is different genes.

To ensure the accuracy of the transcriptome profiling results, we conducted quantitative real-time PCR (qRT-PCR) validation on a subset of differentially expressed genes (Table S4). Five genes—PB.10563, PB.6079, PB.10649, PB.2978, and PB.3758—were randomly selected for this purpose. The expression patterns obtained from qRT-PCR closely paralleled those from the RNA-seq analysis across the different group comparisons (Figure 6). Specifically, both approaches confirmed that PB.10563, PB.6079, PB.10649, and PB.2978 were significantly upregulated, while PB.3758 was consistently downregulated. These findings confirm the reliability and consistency of the transcriptomic data used in this study.

## 4. Discussion

Pubertal molting marks a critical transition in the reproductive cycle of crustaceans, signaling the onset of ovarian development. In the mud crabs, mating is known to accelerate this process dramatically [32,33,48]. Yet, despite its biological importance, the molecular landscape underlying this transition has remained largely unexplored. In this study, we applied full-length transcriptome sequencing to comprehensively map the changes in gene expression and alternative splicing (AS) following mating. Our results offer new insights into the transcriptional profile and signaling pathways that drive post-mating ovarian maturation.

The results of this study indicate that mating exerts a significant impact on multiple molecular processes in the ovary, particularly affecting cell signal transduction, neurochemical pathways, and metabolic networks. These findings suggest that mating may act as a key external stimulus to initially activate ovarian development–related signaling pathways, thereby regulating the supply of materials needed for oocyte growth and subsequent yolk accumulation. Further comparison between the unmated and three days post-mating (UM vs. M3) ovaries revealed substantial remodeling of energy metabolism, especially mitochondrial function and ATP production, reflecting the increased energy demand during rapid ovarian development. In addition, enrichment of the NAFLD pathway suggests potential lipid metabolic dysregulation, possibly associated with fatty acid accumulation. Collectively, these results indicate that mating not only initiates ovarian development through neuroendocrine signaling but also supports oocyte growth and maturation by reorganizing energy and lipid metabolism.

A high-quality transcriptome is essential for investigating dynamic developmental processes like oogenesis. The BUSCO completeness of the reconstructed transcriptome was 25.8%, which was significantly lower than that of the reference genome [36]. This difference likely reflects the tissue-specific and temporally restricted nature of ovarian gene expression [49,50]. Encouragingly, the proportion of fragmented transcripts in our dataset was only 1.9%, which is lower than a previous SMRT-sequencing-based transcriptome (3.2%) [51], highlighting the reliability and accuracy of the sequencing and data processing. The reconstructed transcriptome also revealed substantial isoform diversity, including over 26,000 transcripts not previously annotated—many of which may be novel regulators of ovarian development.

Alternative splicing analysis revealed extensive transcript diversity, with the most frequent AS events being alternative first exon (AF) and alternative 5′ splice site (A5). These splicing patterns were enriched in genes associated with chromatin remodeling, protein regulation, and metabolic processes, suggesting a shift in cellular priorities to support oocyte maturation. It is well known that histone deacetylation inhibits gene expression by removing acetyl groups from active genes. In mice, histone deacetylation occurs rapidly following ovulatory signal induction, indicating its crucial role in ovarian development [52,53,54]. Monoubiquitination of histones is closely associated with the transcriptional regulation of gene expression and the DNA damage response [55]. The presence of such complex isoform architecture implies that AS plays a fundamental role in fine-tuning gene function during ovarian development [56].

Through differential expression and AS analyses, we observed significant transcriptional shifts across developmental stages. The highest number of differentially expressed transcripts (1963) was found in the UM vs. M3 comparison, highlighting how mating triggers a profound molecular reprogramming in the ovary. Several upregulated genes stood out due to their functional importance. For instance, glutathione S-transferase (GST) may reflect increased antioxidant defense during rapid tissue growth [57], ATP synthase supports high energy demands during oocyte proliferation [58,59], and LPIN2-like is likely involved in lipid redistribution and membrane biosynthesis essential for yolk accumulation [58,60,61]. During ovarian development, particularly in the stages of oocyte growth and yolk accumulation, cells required extensive protein synthesis to meet the demands of oocyte development. In the comparative analysis, genes associated with protein synthesis, including EIF3C, EF1a-F2, and EIF3L, were significantly upregulated. This indicates that translation initiation and elongation pathways were markedly activated during the critical stages of ovarian development, thereby enhancing protein synthesis efficiency and providing molecular support for the rapid growth and yolk accumulation of oocytes. These findings further suggested that the regulation of protein synthesis represents one of the central mechanisms underlying ovarian development in crabs.

Pathway enrichment analyses offered even deeper insights. In the UM vs. M1 comparison, GO enrichment pointed to the activation of neurochemical signaling pathways, especially those involving serotonin and catecholamines. These amino acid-derived compounds are known to regulate reproductive hormones in crustaceans and fish alike [62]. For example, serotonin stimulates gonadotropin release in the thoracic ganglia [63], while tyrosine-derived metabolites like dopamine and N-acetylserotonin fluctuate with ovarian stage in Chinese sturgeon, peaking during active vitellogenesis [64]. Among catecholamines, dopamine has been the most extensively studied. During ovarian development, dopamine concentrations generally exhibited a decreasing trend, and numerous studies have confirmed its inhibitory role in oocyte maturation and ovulation [65,66,67]. In contrast, serotonin plays a crucial stimulatory role in regulating oocyte maturation [68]. It is therefore speculated that serotonin and catecholamine signaling pathways may jointly contribute to the neuroendocrine regulation of ovarian development in crabs, and their dynamic changes at different developmental stages may form an “inhibition–activation” molecular switch mechanism that finely regulates the progression of oocyte development. The observed enrichment of these pathways in our data suggests that serotonin and catecholamine signaling may be among the earliest molecular responses to mating, serving as upstream activators of reproductive gene networks [69]. KEGG enrichment analysis showed that mating significantly affected cell signal transduction, neurochemical processes, and metabolic pathways. These changes suggest that mating may initially activate certain ovarian development–related signaling pathways, thereby regulating the provision of materials required for subsequent ovarian development.

In contrast, the UM vs. M3 comparison highlighted enrichment of energy metabolism-related pathways, particularly those associated with mitochondrial function and ATP production, including oxidative phosphorylation, ATP biosynthesis, and electron transport chain activity. These pathways reflect the high energy demands associated with vitellogenesis and oocyte growth [70]. Supporting this, KEGG analysis revealed significant enrichment in pathways like pyruvate metabolism, phenylalanine metabolism, and even non-alcoholic fatty liver disease—systems that are increasingly recognized for their roles in nutrient sensing and energy regulation during reproduction [71]. During ovarian development in *Eriocheir sinensis*, phospholipid metabolism and fatty acid transport pathways were markedly upregulated, indicating a high energy demand for ovarian maturation [72]. In addition, the enrichment of NAFLD suggested a potential metabolic dysregulation in the ovary, which may be related to fatty acid accumulation. This post-mating shift toward bioenergetic activation is consistent with observations in *Portunus trituberculatus*, where energy metabolism genes are downregulated in later stages, likely indicating a transition from energy storage to energy utilization [73].

An intriguing finding was the significant enhancement of host photosynthetic carbon fixation pathways observed three days after mating (Figure 5D). Although the present study did not directly measure changes in the microbiome, a plausible hypothesis is that this phenomenon may be associated with microbial community restructuring following the mating–molting cycle. In *S. paramamosain*, mating typically occurs shortly after female molting. During molting, the old exoskeleton and its associated microbial communities are shed, creating a “blank slate” for the colonization of new microbes [74]. We speculate that this secondary succession of the microbiome may recruit photosynthetic bacteria capable of carbon fixation. The carbon fixed by these symbionts could be directly assimilated by the host to support energetically demanding reproductive activities, such as vitellogenesis and embryonic development. This hypothesis proposes a previously unrecognized microbiome-based nutritional symbiosis strategy, linking molting, reproduction, and energy metabolism. Future studies measuring microbiome changes before and after molting and mating, combined with stable isotope tracing, may help to test this hypothesis.

Structural remodeling of the ovary also emerged as a central theme. The extracellular matrix (ECM)-receptor interaction pathway, enriched in our analysis, is critical for follicular development and oocyte differentiation [75]. In crustaceans and other animals, ECM components such as laminin and collagen interact with integrin receptors to mediate key processes like steroidogenesis, cell migration, and structural integrity [76,77]. These interactions activate intracellular pathways—including TGF-β and PI3K-Akt—that orchestrate follicular growth and survival [78,79]. Given the enrichment of these pathways in our dataset, it’s likely that ECM remodeling contributes to the physical restructuring of the ovary required for oocyte maturation. We also observed enrichment in the HIF-1 and IL-17 signaling pathways. HIF-1 is known to regulate oxygen availability in growing tissues and has been implicated in modulating oocyte developmental timing and blastocyst viability [80,81]. The IL-17 pathway, though traditionally associated with immune function, has recently been linked to follicular maturation in eels [82,83], and may serve a similar role in *S. paramamosain* by regulating ovarian inflammation and tissue remodeling during maturation.

Lipids are vital during ovarian development, not just as structural components but as key energy reserves for embryos and larvae. In *Scylla olivacea*, fatty acids such as palmitic acid increase significantly during vitellogenesis [84], supporting the hypothesis that lipid mobilization supports later-stage oocyte development. In our study, enrichment of pathways like arachidonic acid metabolism and nitrogen metabolism suggests that similar mechanisms are at play in *S. paramamosain*. The upregulation of LPIN2-like, known to regulate phospholipid and triglyceride synthesis, further supports the role of lipid remodeling during this critical phase.

Finally, oxidative stress management appears essential for successful oogenesis. While reactive oxygen species (ROS) can serve as signaling molecules that promote oocyte maturation [85,86], their overaccumulation can cause cellular damage. Our data revealed strong enrichment in GO terms related to cellular oxidant detoxification, with genes such as SOD2, CAT, GPX4, and GSTO1 being significantly upregulated. These enzymes form the core of the antioxidant defense system and likely help maintain redox balance in the rapidly developing ovarian tissue [87,88]. Reduced antioxidant expression in other species has been linked to disrupted ROS balance and altered oocyte development, suggesting that proper ROS regulation is a conserved feature of reproductive biology.

## 5. Conclusions

This study provides a full-length transcriptomic framework for understanding ovarian development following mating in the mud crab (*Scylla paramamosain*). By integrating differential gene expression and alternative splicing analyses, we found that mating initiates extensive transcriptional reprogramming in the ovary. Early responses were characterized by the activation of serotonin and catecholamine signaling pathways, highlighting their potential roles as upstream regulators of reproductive hormones. As ovarian development progressed, pathways related to oxidative phosphorylation, ATP production, lipid metabolism, and extracellular matrix remodeling became dominant, reflecting the increased energy and structural material requirements of vitellogenesis and oocyte maturation. In addition, the upregulation of antioxidant defense genes suggests that maintaining redox homeostasis is crucial for successful oogenesis. Together, these findings not only deepen our understanding of the molecular regulation of reproduction in *S. paramamosain* but also provide valuable insights that may inform broodstock management and reproductive control strategies in aquaculture.

## Figures and Tables

**Table 1:** The 15 types of class codes and their descriptions.

Class Code	Description
=	complete, exact match of intron chain
c	contained in reference (intron compatible)
k	containment of reference (reverse containment)
m	retained intron(s), all introns matched or retained
n	retained intron(s), not all introns matched/covered
j	multi-exon with at least one junction match
e	single exon transfrag partially covering an intron, possible pre-mRNA fragment
o	other same strand overlap with reference exons
s	intron match on the opposite strand (likely a mapping error)
x	exonic overlap on the opposite strand (like o or e but on the opposite strand)
i	fully contained within a reference intron
y	contains a reference within its intron(s)
p	possible polymerase run-on (no actual overlap)
r	repeat (at least 50% bases soft-masked)
u	none of the above (unknown, intergenic)

**Table 2:** The summary of PacBio sequencing data in ovary.

Types	Numbers of Sequences	Length of Isoforms			N50 ^6^
		Min	Mean	Max	
Subreads	59,566,665	51	1705.6	240,063	2300
CCS ^1^	880,044	97	2222.4	14,561	2721
FL ^2^	717,465	51	2057.4	10,605	2681
FLCN ^3^	713,486	50	2012.4	10,573	2654
HQ ^4^	59,156	51	2074.8	8730	2769
LQ ^5^	36	140	2293.1	8310	3319

^1^ CCS = circular consensus sequence; ^2^ FL = full-length; ^3^ FLNC = full-length-non-chimeric; ^4^ HQ = high-quality isoforms; ^5^ LQ = Low-quality isoforms; ^6^ N50 = 50% of reads are longer than this value.

**Table 3:** The summary of features of transcript isoforms after collapsing redundant isoforms with cDNA cupcake, cogent, and CD-HIT.

Numbers of Transcript Isoforms After Collapsing Redundants				Length of Collapsing Redundant Isoforms			N50 ^1^
Reference Genome	Fake Genome	Unmap-Ped	Merge	Min	Max	Mean	
50,170	1483	25	51,637	82	8730	2056	2761

^1^ N50 = 50% of reads are longer than this value.

**Table 4:** The number of each AS events.

AS Events	A3	A5	AF	AL	MX	RI	SE
Numbers	2400	2676	2983	2298	101	1764	1288

**Table 5:** The summary of DAS and DETs characteristics of the six comparison groups.

Group	DAS				DETs		
	Numbers	Upregulated	Downregulated	Protein-Coding Genes	Numbers	Upregulated	Downregulated
UM vs. BM	34	18	16	30	1032	490	542
UM vs. M1	21	9	12	16	1157	575	582
UM vs. M3	34	16	18	27	1963	1004	959
BM vs. M1	22	10	12	19	975	515	460
BM vs. M3	32	17	15	26	1245	664	581
M1 vs. M3	22	10	12	18	921	457	464

## Data Availability

The RNA-seq date were deposited in the Sequence Read Archive of the National Center for Biotechnology Information (NCBI) with the accession number of PRJNA1306745 and PRJNA1307802.

## Supplementary Materials

The following supporting information can be downloaded at: https://www.mdpi.com/article/10.3390/biology14101362/s1.

## References

[1] Sanda T., Shimizu T., Iwasaki T., Dan S., Hamasaki K. (2022). Effect of Temperature on Survival, Intermolt Period, and Growth of Juveniles of Two Mud Crab Species, *Scylla paramamosain* and *Scylla serrata* (Decapoda: Brachyura: Portunidae), under Laboratory Conditions. Nauplius.

[2] Zhao Y., Waqas W., Cui W., Ye S., Gao W., Zhang Q., Lin Z., Zhu D., Lin F., Ikhwanuddin M. (2025). Comparative Analysis of Embryonic Development and Growth Performance among Two Mud Crab Species and Their Hybrids. Aquaculture.

[3] Ye H., Tao Y., Wang G., Lin Q., Chen X., Li S. (2011). Experimental Nursery Culture of the Mud Crab *Scylla paramamosain* (*Estampador*) in China. Aquac. Int..

[4] Nghia T.T., Wille M., Binh T.C., Thanh H.P., Van Danh N., Sorgeloos P. (2007). Improved Techniques for Rearing Mud Crab *Scylla Paramamosain* (Estampador 1949) Larvae. Aquac. Res..

[5] Fishery Bureau of Ministry of Agriculture of China (2024). China Fishery Statistical Yearbook 2024.

[6] Quinitio E.T., De Pedro J., Parado-Estepa F.D. (2007). Ovarian Maturation Stages of the Mud Crab *Scylla serrata*. Aquac. Res..

[7] Christy J.H. (2011). Timing of Hatching and Release of Larvae by Brachyuran Crabs: Patterns, Adaptive Significance and Control. Integr. Comp. Biol..

[8] Waiho K., Fazhan H., Shahreza M.S., Moh J.H.Z., Noorbaiduri S., Wong L.L., Sinnasamy S., Ikhwanuddin M. (2017). Transcriptome Analysis and Differential Gene Expression on the Testis of Orange Mud Crab, *Scylla olivacea*, during Sexual Maturation. PLoS ONE.

[9] Green B.S., Gardner C., Hochmuth J.D., Linnane A. (2014). Environmental Effects on Fished Lobsters and Crabs. Rev. Fish Biol. Fish..

[10] Esmaeili N., Ma H., Kadri S., Tocher D.R. (2024). Protein and Lipid Nutrition in Crabs. Rev. Aquac..

[11] Colpo K.D., López-Greco L.S. (2018). Dynamics of Energy Reserves and the Cost of Reproduction in Female and Male Fiddler Crabs. Zoology.

[12] Usman M., Li A., Wu D., Qinyan Y., Yi L.X., He G., Lu H. (2024). The Functional Role of lncRNAs as ceRNAs in Both Ovarian Processes and Associated Diseases. Non-Coding RNA Res..

[13] Bao C., Yang Y., Huang H., Ye H. (2018). Inhibitory Role of the Mud Crab Short Neuropeptide F in Vitellogenesis and Oocyte Maturation via Autocrine/paracrine Signaling. Front. Endocrinol.

[14] Islam M.S., Kodama K., Kurokora H. (2010). Ovarian Development of the Mud Crab *Scylla paramamosain* in a Tropical Mangrove Swamps, Thailand. J. Sci. Res..

[15] Ye H., Song P., Ma J., Huang H., Wang G. (2010). Changes in Progesterone Levels and Distribution of Progesterone Receptor during Vitellogenesis in the Female Mud Crab (*Scylla paramamosain*). Mar. Freshw. Behav. Phy..

[16] Sharifian S., Kamrani E., Safaie M., Sharifian S. (2015). Oogenesis and Ovarian Development in the Freshwater Crab *Sodhiana Iranica* (Decapoda: Gecarcinuaidae) from the South of Iran. Tissue Cell.

[17] Fazhan H., Waiho K., Wan Norfaizza W.I., Megat F.H., Ikhwanuddin M. (2017). Inter-Species Mating among Mud Crab Genus *Scylla* in Captivity. Aquaculture.

[18] Koolkalya S., Thapanand T., Tunkijjanujij S., Havanont V., Jutagate T. (2006). Aspects in Spawning Biology and Migration of the Mud Crab *Scylla olivacea* in the Andaman Sea, Thailand. Fish. Manag. Ecol..

[19] McLay C.L., López Greco L.S. (2011). A Hypothesis about the Origin of Sperm Storage in the Eubrachyura, the Effects of Seminal Receptacle Structure on Mating Strategies and the Evolution of Crab Diversity: How Did a Race to Be First Become a Race to Be Last?. Zool. Anz..

[20] McLay C.L., Becker C. (2015). Reproduction in Brachyura. Treatise Zool. Anat. Taxon. Biology. Crustac..

[21] Kulski J.K. (2016). Next-Generation Sequencing—An Overview of the History, Tools, and “Omic” Applications. Next Generation Sequencing—Advances, Applications and Challenges.

[22] Satam H., Joshi K., Mangrolia U., Waghoo S., Zaidi G., Rawool S., Thakare R.P., Banday S., Mishra A.K., Das G. (2023). Next-Generation Sequencing Technology: Current Trends and Advancements. Biology.

[23] Nguyen T.V., Jung H., Rotllant G., Hurwood D., Mather P., Ventura T. (2018). Guidelines for RNA-Seq Projects: Applications and Opportunities in Non-Model Decapod Crustacean Species. Hydrobiologia.

[24] Wang Z., Gerstein M., Snyder M. (2009). RNA-Seq: A Revolutionary Tool for Transcriptomics. Nat. Rev. Genet..

[25] Mykles D.L., Burnett K.G., Durica D.S., Joyce B.L., McCarthy F.M., Schmidt C.J., Stillman J.H. (2016). Resources and Recommendations for Using Transcriptomics to Address Grand Challenges in Comparative Biology. Integr. Comp. Biol..

[26] Pop M., Salzberg S.L. (2008). Bioinformatics Challenges of New Sequencing Technology. Trends Genet..

[27] Wenger A.M., Peluso P., Rowell W.J., Chang P.C., Hall R.J., Concepcion G.T., Ebler J., Fungtammasan A., Kolesnikov A., Olson N.D. (2019). Accurate Circular Consensus Long-Read Sequencing Improves Variant Detection and Assembly of a Human Genome. Nat. Biotechnol..

[28] Zhang D., Li W., Chen Z.-J., Wei F.-G., Liu Y.-L., Gao L.-Z. (2020). SMRT- and Illumina-Based RNA-Seq Analyses Unveil the Ginsinoside Biosynthesis and Transcriptomic Complexity in Panax Notoginseng. Sci. Rep..

[29] Ye S., Yu X., Chen H., Zhang Y., Wu Q., Tan H., Song J., Saqib H.S.A., Farhadi A., Ikhwanuddin M. (2022). Full-Length Transcriptome Reconstruction Reveals the Genetic Mechanisms of Eyestalk Displacement and Its Potential Implications on the Interspecific Hybrid Crab (*Scylla Serrata* ♀ × *S. Paramamosain* ♂). Biology.

[30] Byrne A., Cole C., Volden R., Vollmers C. (2019). Realizing the Potential of Full-Length Transcriptome Sequencing. Philos. T R. Soc. B.

[31] Zhu T., Jin M., Luo J., Yang Y., Li X., Peng H., Shen Y., Zhou Q. (2024). Mating behaviour and cholesterol nutritional strategies promoted ovarian development of female swimming crab (*Portunus trituberculatus*). Br. J. Nutr..

[32] Yu Y., Zhang M., Wang D., Xiang Z., Zhao Z., Cui W., Ye S., Fazhan H., Waiho K., Ikhwanuddin M. (2024). Whole Transcriptome RNA Sequencing Provides Novel Insights into the Molecular Dynamics of Ovarian Development in Mud Crab, *Scylla paramamosain* after Mating. Comp. Biochem. Physiol. Part D Genom. Proteom..

[33] Li W., Li S., Wang X., Chen H.-Y., Hao H., Wang K.-J. (2022). Internal Carbohydrates and Lipids as Reserved Energy Supply in the Pubertal Molt of *Scylla paramamosain*. Aquaculture.

[34] Farhadi A., Shi X., Zhang Y., Zhang Y., Li S., Zheng H., Ikhwanuddin M., Ma H. (2021). A Novel Imprinted Gene (Sp-Pol) With Sex-Specific SNP Locus and Sex-Biased Expression Pattern Provides Insights Into the Gonad Development of Mud Crab (*Scylla paramamosain*). Front. Mar. Sci..

[35] Wang D., Yu Y., Gao W., Xiang Z., Zhao Z., Fazhan H., Waiho K., Ikhwanuddin M., Ma H. (2023). Dynamic Changes Characteristics of the Spermatozoon during the Reproductive Process of Mud Crab (*Scylla paramamosain*): From Spermatophore Formation, Transportation to Dispersion. Aquac. Reports.

[36] Zhang Y., Yuan Y., Zhang M., Yu X., Qiu B., Wu F., Tocher D., Zhang J., Ye S., Cui W. (2024). High-resolution chromosome-level genome of *Scylla paramamosain* provides molecular insights into adaptive evolution in crabs. BMC Biol..

[37] Li H. (2018). Minimap2: Pairwise Alignment for Nucleotide Sequences. Bioinformatics.

[38] Huang Y., Niu B., Gao Y., Fu L., Li W. (2010). CD-HIT Suite: A Web Server for Clustering and Comparing Biological Sequences. Bioinformatics.

[39] Update B. (2021). Novel and Streamlined Workflows along with Broader and Deeper Phylogenetic Coverage for Scoring of Eukaryotic, Prokaryotic, and Viral Genomes. Mol. Biol. Evol..

[40] Kriventseva E.V., Kuznetsov D., Tegenfeldt F., Manni M., Dias R., Simão F.A., Zdobnov E.M. (2019). OrthoDB v10: Sampling the Diversity of Animal, Plant, Fungal, Protist, Bacterial and Viral Genomes for Evolutionary and Functional Annotations of Orthologs. Nucleic Acids Res..

[41] Pertea G., Pertea M. (2020). GFF Utilities: GffRead and GffCompare. F1000Research.

[42] Huerta-Cepas J., Forslund K., Coelho L.P., Szklarczyk D., Jensen L.J., Von Mering C., Bork P. (2017). Fast Genome-Wide Functional Annotation through Orthology Assignment by eggNOG-Mapper. Mol. Biol. Evol..

[43] Trincado J.L., Entizne J.C., Hysenaj G., Singh B., Skalic M., Elliott D.J., Eyras E. (2018). SUPPA2: Fast, Accurate, and Uncertainty-Aware Differential Splicing Analysis across Multiple Conditions. Genome Biol..

[44] Patro R., Duggal G., Love M.I., Irizarry R.A., Kingsford C. (2017). Salmon Provides Fast and Bias-Aware Quantification of Transcript Expression. Nat. Methods.

[45] Love M.I., Huber W., Anders S. (2014). Moderated Estimation of Fold Change and Dispersion for RNA-Seq Data with DESeq2. Genome Biol..

[46] Yu G., Wang L.G., Han Y., He Q.Y. (2012). ClusterProfiler: An R Package for Comparing Biological Themes among Gene Clusters. Omi. A J. Integr. Biol..

[47] Schmittgen T.D., Livak K.J. (2001). Analysis of Relative Gene Expression Data Using Real-Time Quantitative PCR and the 2(-Delta Delta C(T)) Method. Methods.

[48] Long X., Guo Q., Wang X., Francis D.S., Cheng Y., Wu X. (2020). Effects of Fattening Period on Ovarian Development and Nutritional Quality of Adult Female Chinese Mitten Crab *Eriocheir sinensis*. Aquaculture.

[49] Johnson B.R., Atallah J., Plachetzki D.C. (2013). The Importance of Tissue Specificity for RNA-Seq: Highlighting the Errors of Composite Structure Extractions. BMC Genom..

[50] Naumova O.Y., Lee M., Rychkov S.Y., Vlasova N.V., Grigorenko E.L. (2013). Gene Expression in the Human Brain: The Current State of the Study of Specificity and Spatiotemporal Dynamics. Child Dev..

[51] Wan H., Jia X., Zou P., Zhang Z., Wang Y. (2019). The Single-Molecule Long-Read Sequencing of *Scylla paramamosain*. Sci. Rep..

[52] Jin J., Ren P., Li X., Zhang Y., Yang W., Ma Y., Lai M., Yu C., Zhang S., Zhang Y. (2023). Ovulatory signal-triggered chromatin remodeling in ovarian granulosa cells by HDAC2 phosphorylation activation-mediated histone deacetylation. Epigenetics Chromatin.

[53] Wang Z., Zang C., Cui K., Schones D., Barski A., Peng W., Zhao K. (2009). Genome-wide mapping of HATs and HDACs reveals distinct functions in active and inactive genes. Cell.

[54] Gonzalez-Zuniga M., Contreras P.S., Estrada L.D., Chamorro D., Villagra A., Zanlungo S., Seto E., Alvarez A.R. (2014). c-Abl stabilizes HDAC2 levels by tyrosine phosphorylation repressing neuronal gene expression in Alzheimer’s disease. Mol. Cell.

[55] Cole A.J., Clifton-Bligh R., Marsh D.J. (2015). Histone H2B monoubiquitination: Roles to play in human malignancy. Endocr. Relat. Cancer.

[56] Dioken D.N., Ozgul I., Erson-Bensan A.E. (2025). The 3′ End of the Tale—Neglected Isoforms in Cancer. FEBS Lett..

[57] Ma M., Zhang Y.X., Chen D., Smagghe G., Wang J.J., Wei D. (2022). Functional Characterization of a Glutathione S-Transferase Gene GSTe10 That Contributes to Ovarian Development in *Bactrocera dorsalis* (Hendel). Entomol. Gen..

[58] Wang T., Wang T., Zhang M., Shi X., Zhang M., Wang H., Yang X., Yu Z., Liu J. (2021). The Ovarian Development Genes of Bisexual and Parthenogenetic Haemaphysalis Longicornis Evaluated by Transcriptomics and Proteomics. Front. Vet. Sci..

[59] Moreira H.N.S., Barcelos R.M., Vidigal P.M.P., Klein R.C., Montandon C.E., Maciel T.E.F., Carrizo J.F.A., Costa de Lima P.H., Soares A.C., Martins M.M. (2017). A Deep Insight into the Whole Transcriptome of Midguts, Ovaries and Salivary Glands of the *Amblyomma sculptum* Tick. Parasitol. Int..

[60] Trapp J., Almunia C., Gaillard J.C., Pible O., Chaumot A., Geffard O., Armengaud J. (2016). Proteogenomic Insights into the Core-Proteome of Female Reproductive Tissues from Crustacean Amphipods. J. Proteomics.

[61] Yepiz-Plascencia G., Vargas-Albores F., Higuera-Ciapara I. (2000). Penaeid Shrimp Hemolymph Lipoproteins. Aquaculture.

[62] Barcellos L.J.G., Volpato G.L., Barreto R.E., Coldebella I., Ferreira D. (2011). Chemical Communication of Handling Stress in Fish. Physiol. Behav..

[63] Kulkarni G.K., Nagabhushanam R., Amaldoss G., Jaiswal R.G., Fingerman M. (1992). In Vivo Stimulation of Ovarian Development in the Red Swamp Crayfish, *Procambarus clarkii* (Girard), by 5-Hydroxytryptamine. Invertebr. Reprod. Dev..

[64] Zhu Y., Wu J., Leng X., Du H., Wu J., He S., Luo J., Liang X., Liu H., Wei Q. (2020). Metabolomics and Gene Expressions Revealed the Metabolic Changes of Lipid and Amino Acids and the Related Energetic Mechanism in Response to Ovary Development of Chinese Sturgeon (*Acipenser sinensis*). PLoS ONE.

[65] Aizen J., Meiri I., Tzchori I., Levavi-Sivan B., Rosenfeld H. (2005). Enhancing spawning in the grey mullet (*Mugil cephalus*) by removal of dopaminergic inhibition. Gen. Comp. Endocrinol..

[66] Vidal B., Pasqualini C., Le Belle N., HHolland M., Sbaihi M., Vernier P., Zohar Y., Dufour S. (2004). Dopamine inhibits luteinizing hormone synthesis and release in the juvenile *European eel*: A neuroendocrine lock for the onset of puberty. Biol. Reprop..

[67] Yaron Z. (1995). Endocrine control of gametogenesis and spawning induction in the carp. Aquaculture.

[68] Tinikul Y., Mercier A.J., Soonklang N., Sobhon P. (2008). Changes in the levels of serotonin and dopamine in the central nervous system and ovary, and their possible roles in the ovarian development in the giant freshwater prawn, *Macrobrachium rosenbergii*. Gen. Comp. Endocrinol..

[69] Esmaeili N., Kadri S., Kumar V., Ma H. (2025). Serotonin: A Multifunctional Molecule in Crustaceans. Rev. Aquac..

[70] Kleppe L., Edvardsen R.B., Furmanek T., Taranger G.L., Wargelius A. (2014). Global Transcriptome Analysis Identifies Regulated Transcripts and Pathways Activated during Oogenesis and Early Embryogenesis in Atlantic Cod. Mol. Reprod. Dev..

[71] Inigo M., Deja S., Burgess S.C. (2021). Ins and Outs of the TCA Cycle: The Central Role of Anaplerosis. Annu. Rev. Nutr..

[72] Feng Q., Liu M., Cheng Y., Wu X. (2022). Comparative transcriptome analysis reveals the process of ovarian development and nutrition metabolism in Chinese mitten crab, Eriocheir sinensis. Front. Genet..

[73] Zhang Y., Chen Y., Hou C., Wang C., Mu C. (2025). Analysis of cDNA microarrays revealed the effects of mating on the ovary and hepatopancreas of female swimming crab (*Portunus trituberculatus*) during the late stage of ovarian develoment. Comp. Biochem. Phys. D.

[74] Zhang M., Zhang X., Tran N.T., Sun Z., Zhang X., Ye H., Zhang Y., Ma H., Aweya J.J., Li S. (2021). Molting alters the microbiome, immune response, and digestive enzyme activity in mud crab (*Scylla paramamosain*). Msystems.

[75] Nagyová E., Němcová L., Camaioni A. (2022). Cumulus Extracellular Matrix Is an Important Part of Oocyte Microenvironment in Ovarian Follicles: Its Remodeling and Proteolytic Degradation. Int. J. Mol. Sci..

[76] Curry T.E., Osteen K.G. (2003). The Matrix Metalloproteinase System: Changes, Regulation, and Impact throughout the Ovarian and Uterine Reproductive Cycle. Endocr. Rev..

[77] Dong Y., Lyu L., Zhang D., Li J., Wen H., Shi B. (2021). Integrated lncRNA and mRNA Transcriptome Analyses in the Ovary of Cynoglossus Semilaevis Reveal Genes and Pathways Potentially Involved in Reproduction. Front. Genet..

[78] Kreeger P.K., Deck J.W., Woodruff T.K., Shea L.D. (2006). The in Vitro Regulation of Ovarian Follicle Development Using Alginate-Extracellular Matrix Gels. Biomaterials.

[79] Li Z., Tan S., Qi L., Chen Y., Liu H., Liu X., Sha Z. (2023). Genome-Wide Characterization of Integrin (ITG) Gene Family and Their Expression Profiling in Half-Smooth Tongue Sole (*Cynoglossus semilaevis*) upon *Vibrio anguillarum* Infection. Comp. Biochem. Phys. D.

[80] Song H., Zhu B., Dong T., Wang W., Hu M., Yan X., Xu S., Hu H. (2023). Whole-Genome Resequencing Reveals Selection Signatures for Caviar Yield in Russian Sturgeon (*Acipenser gueldenstaedtii*). Aquaculture.

[81] Turhan A., Pereira M.T., Schuler G., Bleul U., Kowalewski M.P. (2021). Hypoxia-Inducible Factor (HIF1alpha) Inhibition Modulates Cumulus Cell Function and Affects Bovine Oocyte Maturation In Vitro. Biol. Reprod..

[82] Lai X., Peng S., Feng J., Zou P., Wang Y. (2022). Immune Function Modulation during Artificial Ovarian Maturation in Japanese Eel (*Anguilla japonica*): A Transcriptome Profiling Approach. Fish Shellfish Immunol..

[83] Lai X.J., Peng S., Wang Y.L. (2022). Dynamic Transcriptome Analysis of Ovarian Follicles in Artificial Maturing Japanese Eel (*Anguilla japonica*). Theriogenology.

[84] Ghazali A., Azra M.N., Noordin N.M., Abol-Munafi A.B., Ikhwanuddin M. (2017). Ovarian Morphological Development and Fatty Acids Profile of Mud Crab (*Scylla olivacea*) Fed with Various Diets. Aquaculture.

[85] Arenas-Ríos E., León-Galván M.A., Mercado P.E., López-Wilchis R., Cervantes D.L.M.I., Rosado A. (2007). Superoxide Dismutase, Catalase, and Glutathione Peroxidase in the Testis of the Mexican Big-Eared Bat (*Corynorhinus mexicanus*) during Its Annual Reproductive Cycle. Comp. Biochem. Phys. A.

[86] Behrman H.R., Kodaman P.H., Preston S.L., Gao S. (2001). Oxidative Stress and the Ovary. J. Soc. Gynecol. Investig..

[87] Lu J., Wang Z., Cao J., Chen Y., Dong Y. (2018). A Novel and Compact Review on the Role of Oxidative Stress in Female Reproduction. Reprod. Biol. Endocrinol..

[88] Zhang Y., Li Y.-D., Yang Q.-B., Jiang S., Jiang S.-G., Zhou F.-L. (2022). Transcriptome Analysis of *Metapenaeus affinis* Reveals Genes Involved in Gonadal Development. ISR J. Aquac. Bamidgeh.

